# Outcomes of EKOS (Ultrasound-Assisted Thrombolysis) for Intermediate–High-Risk Pulmonary Embolism: A PERT-Guided Cohort Study

**DOI:** 10.1093/icvts/ivag168

**Published:** 2026-06-02

**Authors:** Mohammed N H E Ali, Rashad Zayat, Nima Hatam, Johannes Greven, Jasper Nies, Ahmed F A Mohammed, Alexander Kersten, Nikolaus Marx, Michael Dreher, Yusuf Shieba, Jan Spillner, Sebastian Kalverkamp

**Affiliations:** Department of Cardiac Surgery, Faculty of Medicine, RWTH University Hospital, Aachen 52074, Germany; Department of Cardiac Surgery, Faculty of Medicine, RWTH University Hospital, Aachen 52074, Germany; Department of Cardiac Surgery, Faculty of Medicine, RWTH University Hospital, Aachen 52074, Germany; Department of Thoracic Surgery, Faculty of Medicine, RWTH University Hospital, Aachen 52074, Germany; Department of Thoracic Surgery, Faculty of Medicine, RWTH University Hospital, Aachen 52074, Germany; Department of Cardiac Surgery, Faculty of Medicine, RWTH University Hospital, Aachen 52074, Germany; Department of Cardiothoracic Surgery, Faculty of Medicine, Qena University, Qena, Egypt; Department of Cardiology, Pneumology, Angiology, and Intensive Care, Faculty of Medicine, RWTH University Hospital, Aachen 52074, Germany; Department of Cardiology, Pneumology, Angiology, and Intensive Care, Faculty of Medicine, RWTH University Hospital, Aachen 52074, Germany; Department of Pneumology and Intensive Care Medicine, University Hospital RWTH Aachen, Aachen 52074, Germany; Department of Cardiac Surgery, Faculty of Medicine, RWTH University Hospital, Aachen 52074, Germany; Department of Cardiothoracic Surgery, Faculty of Medicine, Qena University, Qena, Egypt; Department of Thoracic Surgery, Faculty of Medicine, RWTH University Hospital, Aachen 52074, Germany; Department of Thoracic Surgery, Faculty of Medicine, RWTH University Hospital, Aachen 52074, Germany

**Keywords:** pulmonary embolism, catheter-directed thrombolysis, EKOS, pulmonary embolism response team, right-ventricular dysfunction

## Abstract

**Objectives:**

Acute intermediate–high-risk pulmonary embolism (PE) is associated with right-ventricular (RV) dysfunction, biomarker elevation, and risk of early clinical deterioration. Ultrasound-assisted catheter-directed thrombolysis (USAT) using the Eko Sonic Endovascular System (EKOS) system is increasingly used in selected patients; however, real-world data on physiological response and clinically relevant in-hospital outcomes remain limited.

**Methods:**

We conducted a retrospective single-centre analysis of 128 consecutive patients with acute intermediate–high-risk PE treated with EKOS-guided USAT within a structured Pulmonary Embolism Response Team (PERT) pathway between August 2017 and December 2023. Changes in RV/LV ratio, B-type natriuretic peptide (BNP), and cardiac troponin before and after intervention were assessed. A composite end-point of death, extracorporeal membrane oxygenation (ECMO), renal replacement therapy, or invasive mechanical ventilation captured clinically meaningful deterioration. Multivariable logistic regression identified determinants of the composite end-point.

**Results:**

RV/LV ratio and biomarkers improved significantly after intervention. Median RV/LV ratio decreased from 1.6 to 1.0, and BNP declined from 2365 to 877 pg/mL (both *P* < .001). Clinically meaningful in-hospital deterioration requiring escalation of organ support occurred in a subset of patients and was predominantly associated with baseline disease severity. Short-term survival remained favourable (60-day probability 96.9%).

**Conclusions:**

In this real-world, PERT-guided cohort, EKOS was associated with rapid physiological improvement and favourable short-term survival in patients with intermediate–high-risk PE. These findings should be interpreted as descriptive single-centre experience.

## Graphical Abstract

**Figure:**
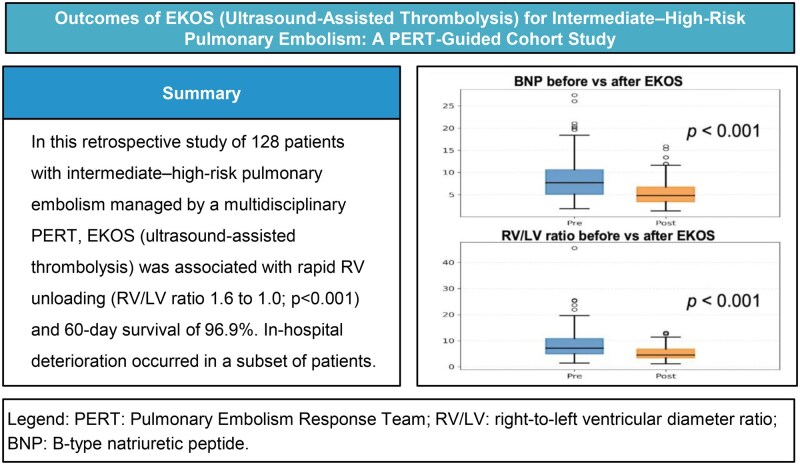

## INTRODUCTION

Acute pulmonary embolism (PE) remains a major cause of cardiovascular morbidity and mortality. Early outcome is largely determined by the severity of right-ventricular (RV) dysfunction and haemodynamic compromise; therefore, contemporary risk stratification integrates clinical severity indices, imaging markers of RV function, and cardiac biomarkers to guide monitoring and reperfusion strategies.^1^^,^^2^

Systemic thrombolysis can rapidly reduce thrombus burden in high-risk PE but is limited by substantial bleeding risk.^1^ Consequently, catheter-based reperfusion strategies have been increasingly adopted in selected patients.^3^^,^^4^ Ultrasound-assisted catheter-directed thrombolysis (USAT) using the Eko Sonic Endovascular System (EKOS) system, combines low-dose thrombolytic infusion with ultrasound energy to facilitate intrathrombus drug penetration.^3^^,^^5^

Previous studies, including ULTIMA, SEATTLE II, and OPTALYSE PE, have demonstrated improvements in RV unloading and short-term physiological parameters.^6–8^

However, real-world data remain limited, particularly regarding escalation pathways, organ dysfunction, and clinically meaningful in-hospital deterioration.^9–11^ In addition, complex PE presentations may require individualized treatment selection, including catheter-based reperfusion, systemic thrombolysis, or surgical embolectomy depending on haemodynamic status, anatomical features, and bleeding risk.^12^

Accordingly, the present study aimed to provide a descriptive real-world evaluation of patients with intermediate–high-risk PE treated with EKOS within a structured Pulmonary Embolism Response Team (PERT) pathway, focusing on physiological response, in-hospital outcomes, and determinants of clinical deterioration.

## METHODS

### Study design and patient population

We conducted a retrospective single-centre cohort study of consecutive adult patients with acute intermediate–high-risk PE treated with USAT using the EKOS system between August 2017 and December 2023 at RWTH Aachen University Hospital. Intermediate–high-risk PE was defined according to contemporary European Society of Cardiology criteria, including evidence of RV dysfunction on imaging and/or elevated cardiac biomarkers in the absence of sustained haemodynamic instability.^2^

The study was approved by the local ethics committee of RWTH University Hospital (EK 25–286). The requirement for informed consent was waived because of the retrospective observational design and use of routinely collected clinical data. No biological material was collected or stored for research purposes, and no research biobank or data repository for multiple or indefinite future use was established. Therefore, the provisions of the WMA Declaration of Taipei regarding research databases and biobanks were not applicable.

### Pulmonary embolism response team

**Figure 1.**
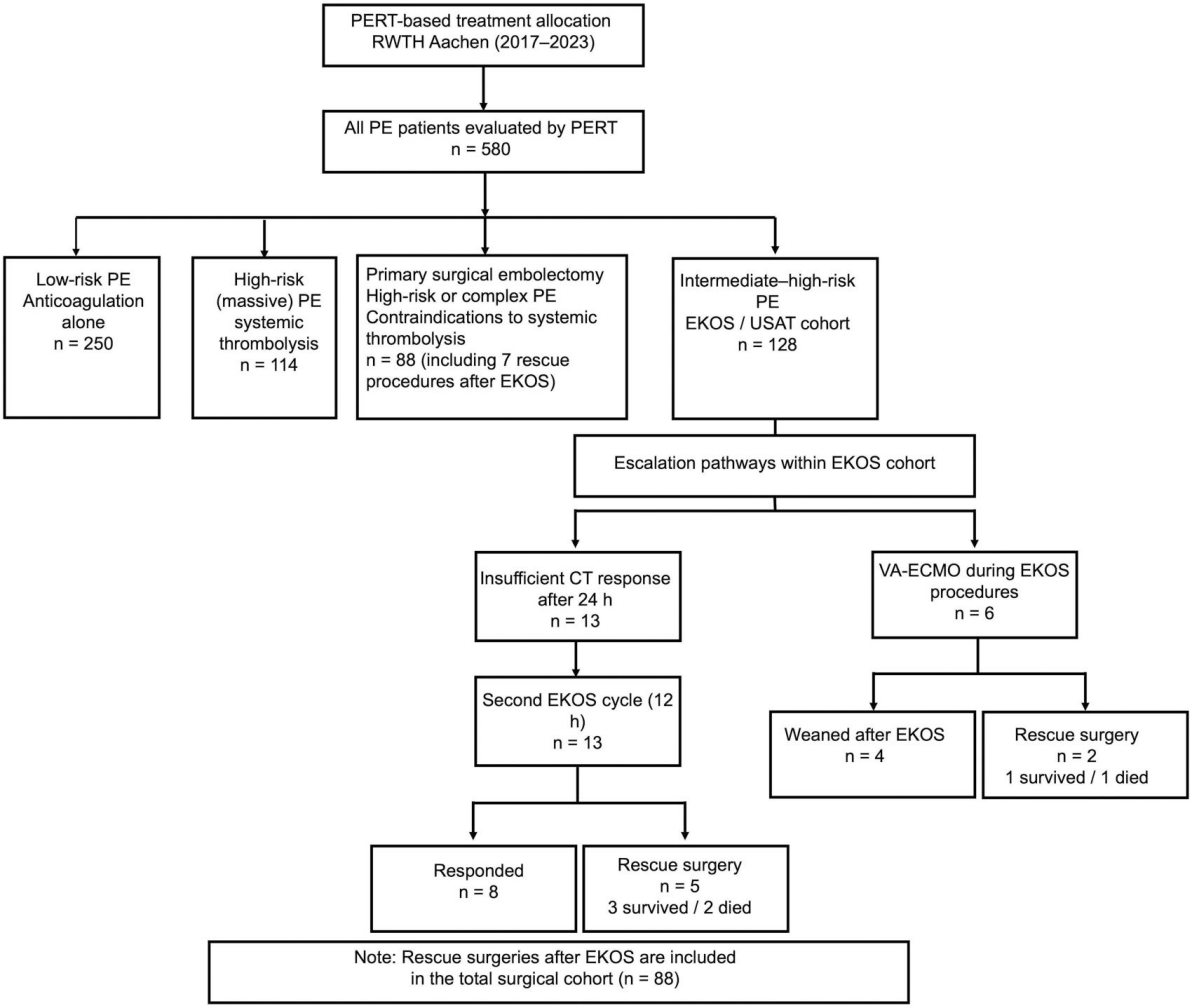
PERT-Based Treatment Allocation of Pulmonary Embolism Patients at RWTH Aachen University Hospital (2017-2023). The flowchart shows risk-stratified decision-making among 580 consecutive PE patients evaluated by the PERT, with allocation to anticoagulation alone (*n* = 250), systemic thrombolysis (*n* = 114), primary surgical embolectomy (*n* = 88, including 7 rescue procedures after EKOS), and EKOS-based ultrasound-assisted thrombolysis (*n* = 128). Escalation pathways within the EKOS cohort are detailed. Note: Rescue surgeries after EKOS are included in the total surgical cohort (*n* = 88).

All patients were evaluated and managed within structured a 24/7. Pulmonary Embolism Response Team pathway. At RWTH Aachen, a dedicated hotline is carried by the on-duty cardiologist in the intensive care unit. Following CT confirmation of PE, cases are reviewed using imaging findings, transthoracic echocardiography, and cardiac biomarkers, including B-type natriuretic peptide (BNP) and troponin. The multidisciplinary PERT comprises specialists from interventional cardiology, cardiac surgery, thoracic surgery, cardioanesthesiology, and pneumology. Treatment decisions, including escalation to reperfusion therapy, were made by consensus, integrating haemodynamic status, RV dysfunction, biomarker profile, bleeding risk, and comorbidities.^13–15^ An overview of the institutional PERT-based treatment allocation algorithm during the study period is provided in **Figure 1**.

### PERT treatment allocation algorithm

All patients were managed according to a risk-stratified institutional algorithm driven by haemodynamic stability, bleeding risk, and clot anatomy. Low-risk patients received anticoagulation alone. Intermediate–high-risk patients with RV dysfunction but preserved haemodynamic stability were considered for EKOS. High-risk (massive) PE or patients with contraindications to thrombolysis were treated with systemic thrombolysis or surgical embolectomy. During the study period (2017-2023), large-bore mechanical thrombectomy was not performed at our centre, and catheter-based reperfusion, when selected, was performed exclusively using the EKOS system **Figure 1**.

### EKOS procedure

Ultrasound-assisted catheter-directed thrombolysis was performed using the EKOS Endovascular System (Boston Scientific). Catheters were positioned in the affected pulmonary arteries under fluoroscopic guidance. Alteplase was infused at 1 mg/h per catheter, with bilateral placement when appropriate, for a total dose of up to 24 mg over 12-24 h. Concomitant anticoagulation with unfractionated heparin was maintained according to institutional standards.

Before EKOS catheter removal, all patients underwent repeat CT angiography to reassess thrombus burden. If CT showed sufficient thrombus reduction with improvement or normalization of RV/LV ratio, the catheter was removed. In cases of insufficient response after the initial 24-h treatment cycle, therapy was extended with a second 12-h EKOS cycle followed by repeat reassessment. No patient underwent systemic thrombolysis as bailout after EKOS.

### Assessment of physiological response

Physiological response was evaluated using imaging and biomarker parameters obtained at baseline and after intervention during index hospitalization. Baseline RV/LV ratio, BNP, and troponin were defined as the last available measurements before EKOS initiation. Post-intervention values were defined as the first available reassessment after completion of the initial EKOS cycle or, where applicable, after the extended second cycle. RV/LV ratio was assessed by computed tomography pulmonary angiography and/or transthoracic echocardiography, according to clinical availability. Cardiac biomarkers, including BNP and cardiac troponin, were measured using standard institutional assays. RV/LV ratio and biomarker trajectories were analysed as indicators of RV unloading and myocardial stress.^2^^,^^6^^,^^7^

### Outcomes and definitions

Short-term survival was assessed using all-cause mortality up to 60 days. In-hospital clinical deterioration was captured using a prespecified composite end-point comprising death, extracorporeal Membrane Oxygenation (ECMO), renal replacement therapy, or invasive mechanical ventilation from EKOS initiation until hospital discharge or death. Additional safety and organ dysfunction outcomes included acute kidney failure, respiratory failure, haemorrhagic anaemia, and other in-hospital complications, defined according to institutional clinical criteria.

Non-response to EKOS was defined clinically as insufficient reduction of RV overload on follow-up imaging and/or clinical deterioration, including persistent or worsening RV failure, worsening oxygenation, acidosis, or ongoing haemodynamic compromise. New-onset invasive mechanical ventilation referred to initiation after EKOS in previously non-ventilated patients.

All extracorporeal support used in this study consisted of veno-arterial extracorporeal membrane oxygenation (VA-ECMO), typically via femoro–femoral cannulation.

### Statistical analysis

Continuous variables are reported as median with interquartile range, and categorical variables as counts and percentages. Pre- and post-interventional physiological parameters were compared using paired non-parametric tests, as appropriate. Survival was analysed using Kaplan–Meier methods. Univariable and multivariable logistic regression analyses were performed to identify predictors of the composite deterioration end-point. A sensitivity subanalysis was performed excluding patients with pre-intervention shock, mechanical ventilation, acute kidney failure, or mechanical circulatory support to assess the influence of baseline critical illness on post-interventional adverse outcomes. Variables entered into the regression analysis were selected based on clinical relevance and event frequency. Univariable analyses were performed for candidate baseline variables, and a parsimonious multivariable logistic regression model was constructed to identify independent predictors of the composite deterioration end-point. Statistical analyses were performed using R (version 4.5.2), and a two-sided *P*-value < .05 was considered statistically significant.

## RESULTS

### Study population and baseline characteristics

Of 580 patients evaluated within the institutional PERT pathway during the study period, 128 underwent EKOS-based therapy and comprised the study cohort (**Figure 1**). Baseline characteristics, comorbidities, and initial haemodynamic status are summarized in **Table 1**. The median age of the cohort was 65.5 years (IQR 54.8-74.0), and 35.2% of patients were female. According to the PE Severity Index, most patients were classified as low or intermediate risk, while 4.7% met criteria for high-risk PE. Evidence of RV strain was common, with a median pre-intervention RV/LV ratio of 1.6 (IQR 1.4-1.7) and elevated baseline BNP levels (median 2365 pg/mL, IQR 1153-3656). Prior to intervention, 16.4% of patients required vasopressor support and 17.2% required invasive mechanical ventilation.

**Table 1.** Baseline Clinical Characteristics, Comorbidities, Biomarker Values, Haemodynamic Instability Markers, and Imaging Findings Prior to Initiation of USAT

Characteristic	Value
Demographics	
Age, years	65.5 (54.8-74.0)
Female sex	45 (35.2%)
PESI	
PESI low	61 (47.7%)
PESI intermediate	61 (47.7%)
PESI high	6 (4.7%)
Right-Heart Strain and Imaging	
RV/LV ratio (CT), pre-intervention	1.6 (1.4-1.7)
Clinical signs of right-heart failure	75 (58.6%)
Biomarkers	
Troponin, pg/mL	49.0 (24.0-114.5)
NT-proBNP, pg/mL	2800.0 (1400.0-5250.0)
AST, U/L	30.5 (23.8-50.0)
ALT, U/L	29.0 (17.8-55.0)
Haemodynamic Instability Before EKOS	
Any pre-shock	6 (4.7%)
Obstructive shock	6 (4.7%)
Septic shock	1 (0.8%)
Haemorrhagic shock	0 (0.0%)
Vasopressor use	21 (16.4%)
Any invasive organ support	27 (21.1%)
Mechanical ventilation	22 (17.2%)
Pre-intervention VA-ECMO	1 (0.8%)
Comorbidities	
Acute kidney failure	19 (14.8%)
Chronic kidney disease	10 (7.8%)
Arterial hypertension	17 (13.3%)
Coronary artery disease	4 (3.1%)
Left-heart failure	10 (7.8%)
COPD	2 (1.6%)
Diabetes mellitus type 2	18 (14.1%)
Cancer history	11 (8.6%)
Obesity	32 (25.0%)
Deep vein thrombosis	67 (52.3%)
Other Clinical Findings	
Pleural effusion	20 (15.6%)
Acute respiratory insufficiency	62 (48.4%)
Central venous catheter present	16 (12.5%)
Bacterial infection	0 (0.0%)
Staphylococcus sepsis	1 (0.8%)
Candidemia	1 (0.8%)
E. coli infection	6 (4.7%)

Abbreviations: ALT = alanine aminotransferase; AST = aspartate aminotransferase; BNP = B-type natriuretic peptide; COPD = chronic obstructive pulmonary disease; CT = computed tomography; ECMO = extracorporeal membrane oxygenation; EKOS = EkoSonic Endovascular System; NT-proBNP = N-terminal pro–B-type natriuretic peptide; PESI = Pulmonary Embolism Severity Index; RV/LV = right ventricular to left ventricular diameter ratio; VA-ECMO = veno-arterial extracorporeal membrane oxygenation.

### Haemodynamic and biomarker response

**Figure 2.**
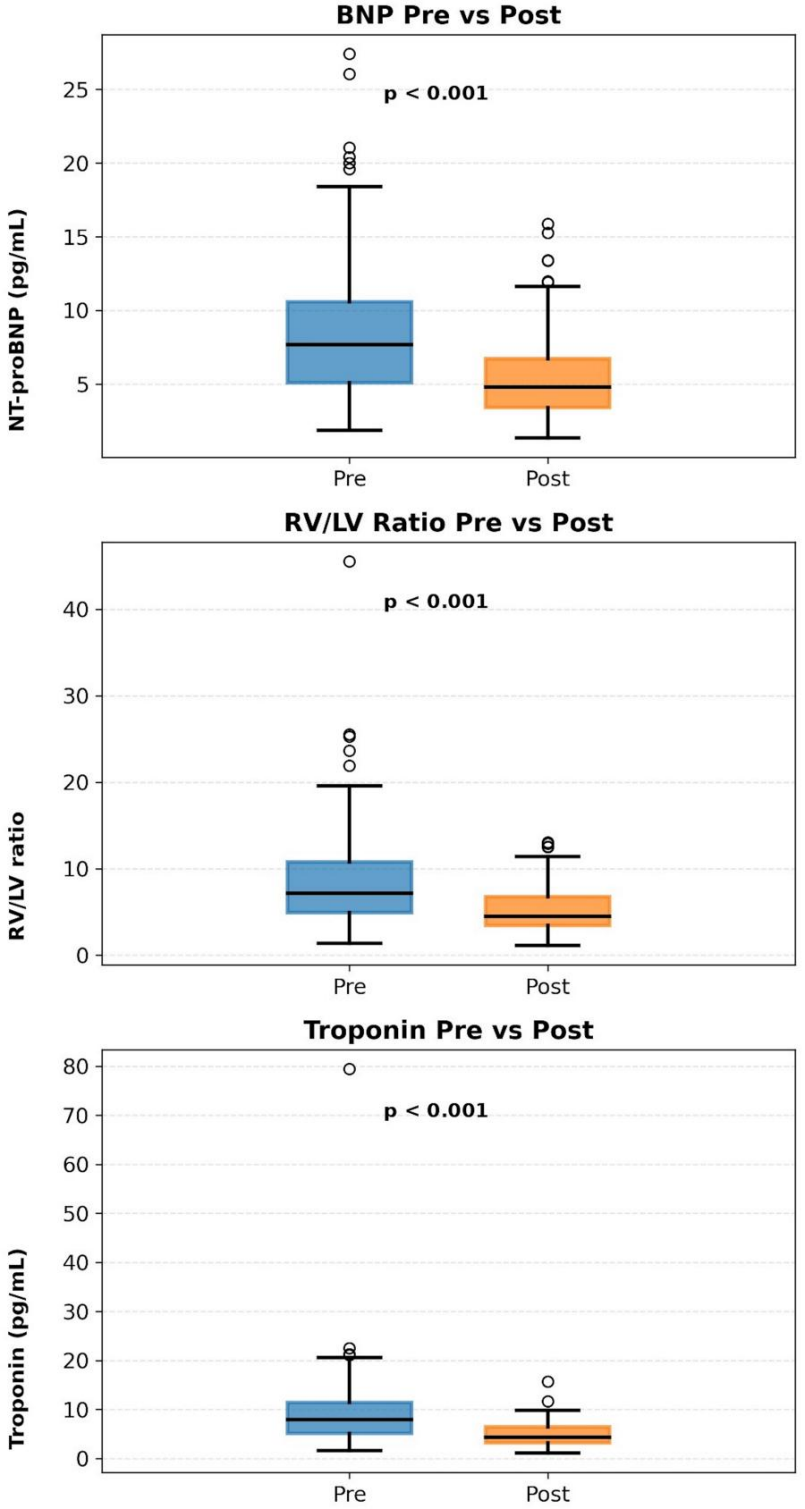
Biomarker and Imaging Response Following EKOS-Based USAT. Boxplots illustrating changes in BNP, right ventricular to left ventricular diameter ratio (RV/LV), and cardiac troponin before and after USAT. Abbreviations: BNP = B-type natriuretic peptide; RV/LV = right ventricular to left ventricular diameter ratio; TrT = cardiac troponin T.

Post-procedural haemodynamic and biomarker outcomes are detailed in **Table 2**. The median postintervention RV/LV ratio decreased to 1.0 (IQR 0.9-1.1), and median BNP declined to 877 pg/mL (IQR 618-1264). Paired analyses confirmed a significant response (**Figure 2**). The RV/LV ratio decreased by a median of −0.50 (*P* < .001), BNP levels decreased by a median of −1303 pg/mL (*P* < .001), and troponin levels declined by a median of −24 pg/mL (*P* < .001), indicating rapid RV unloading.

**Table 2.** Paired Haemodynamic and Biomarker Changes Before and After EKOS Therapy

Parameter	*N*	Pre, median (IQR)	Post, median (IQR)	Δ median (post–pre)	*P*-value (Wilcoxon)
RV/LV ratio	125	1.6 (1.4-1.7)	1.0 (0.9-1.1)	−0.5	<.001
BNP, pg/mL	124	2365.0 (1217.5-3656.0)	877.0 (618.2-1263.5)	−1303.0	<.001
Troponin, pg/mL	124	82.0 (44.0-152.2)	56.0 (33.0-98.0)	−24.0	<.001

Paired comparisons were conducted using the Wilcoxon signed-rank test. RV/LV ratio was assessed by echocardiography. BNP—B-type natriuretic peptide; EKOS—EkoSonic Endovascular System; IQR—interquartile range; RV/LV—right ventricular to left ventricular diameter ratio.

### Organ dysfunction and safety outcomes

Organ dysfunction and safety outcomes during index hospitalization are summarized in **Table 3**. Acute respiratory insufficiency was present in 48.4% of patients at baseline and remained frequent after intervention (49.2%), reflecting persistent preexisting respiratory failure rather than new-onset deterioration. Only one patient (0.8%) developed new respiratory insufficiency following EKOS therapy. Similarly, acute kidney failure was present in 14.8% at baseline and did not increase after intervention, indicating no excess renal toxicity associated with the procedure. Other post-interventional adverse events included acute haemorrhagic anaemia (21.9%), cardiac arrest (7.8%), ECMO use (4.7%), and renal replacement therapy (5.5%).

**Table 3.** Post-Intervention Haemodynamic, Biomarker, and Clinical Outcomes following USAT (EKOS)

Outcome	Value
Post-procedural RV/LV ratio	1.0 (0.9-1.1)
Post-procedural BNP, pg/mL	877.0 (618.2-1263.5)
Post-procedural troponin, pg/mL	56.0 (33.0-98.0)
Length of hospital stay, days	9.0 (6.0-15.0)
Post-intervention shock and haemodynamic support, *n* (%)	
Any post-procedural shock	11 (8.6%)
Obstructive shock	5 (3.9%)
Septic shock	1 (0.8%)
Haemorrhagic shock	5 (3.9%)
Vasopressor use	21 (16.4%)
Advanced organ support and complications, *n* (%)	
VA-ECMO	6 (4.7%)
Renal replacement therapy (dialysis)	7 (5.5%)
Acute kidney failure	19 (14.8%)
Acute haemorrhagic anaemia	28 (21.9%)
Central venous catheter in situ	16 (12.5%)
Cardiac arrest	10 (7.8%)
Acute respiratory insufficiency	63 (49.2%)
Infarct pneumonia	20 (15.6%)

RV/LV ratio was assessed by echocardiography; pre-intervention CT was available in all patients. Acute kidney failure, haemorrhagic anaemia, and respiratory insufficiency reflect complications during the index hospitalization. Abbreviations: BNP = B-type natriuretic peptide; CT = computed tomography; EKOS = EkoSonic Endovascular System; RV/LV = right ventricular to left ventricular diameter ratio; VA-ECMO = veno-arterial extracorporeal membrane oxygenation.

### Sensitivity subanalysis

To evaluate the influence of baseline critical illness on adverse outcomes, a sensitivity analysis excluding patients with pre-intervention shock, mechanical ventilation, acute kidney failure, or pre-intervention mechanical circulatory support was performed (*n* = 96; **Table S1**). In this sub-cohort, no cases of ECMO, dialysis, or new-onset acute kidney failure were observed. Acute haemorrhagic anaemia occurred in 11.5%, cardiac arrest in 3.1%, and acute respiratory insufficiency in 44.8% of patients. This suggests that escalation of organ support was largely driven by baseline severity.

### Composite clinical endpoint and predictors

Overall, 23 patients (18.0%) met the prespecified composite clinical end-point of death, ECMO, renal replacement therapy, or invasive mechanical ventilation during index hospitalization (**Table 3**). In univariable analyses, PESI high-risk category, left heart failure, baseline troponin, and acute kidney failure were associated with the composite end-point. In a parsimonious multivariable logistic regression model (**Table 4**), high-risk PE Severity Index classification was independently associated with the composite end-point (adjusted odds ratio [OR] 11.14, 95% confidence interval [CI] 1.61-77.29; *P* = .015). Acute kidney failure was also independently associated with a markedly increased risk of adverse outcome (adjusted OR 11.23, 95% CI 3.61-34.92; *P* < .001).

**Table 4.** Univariable and Multivariable Predictors of the Composite Clinical Endpoint After EKOS Therapy

Predictor	Univariable OR (95% CI)	*P*-value	Multivariable OR (95% CI)	*P*-value
Age	0.99 (0.96-1.02)	.453	NA	NA
Female sex	0.98 (0.38-2.53)	.967	NA	NA
PESI high-risk category	10.84 (1.85-63.43)	.008	11.14 (1.61-77.29)	.015
RV dysfunction on CT	1.80 (0.70-4.62)	.219	NA	NA
RV dysfunction on echocardiography	0.90 (0.24-3.41)	.877	NA	NA
Left heart failure	5.56 (1.46-21.16)	.012	NA	NA
RV/LV ratio (pre)	1.15 (0.24-5.56)	.861	NA	NA
BNP (pre, log-transformed)	1.55 (0.94-2.58)	.089	NA	NA
Troponin (pre, log-transformed)	1.69 (1.06-2.69)	.029	NA	NA
Acute kidney failure	11.11 (3.74-33.07)	<.001	11.23 (3.61-34.92)	<.001

Multivariable logistic regression assessing predictors of a composite clinical end-point defined as death, ECMO, renal replacement therapy, or invasive mechanical ventilation during index hospitalization. Variables were selected based on clinical relevance and event frequency. Odds ratios represent adjusted estimates. Abbreviations: CI = confidence interval; CT = computed tomography; BNP = B-type natriuretic peptide; ECMO = extracorporeal membrane oxygenation; EKOS = EkoSonic Endovascular System; NA = not applicable; OR = odds ratio; PESI = Pulmonary Embolism Severity Index; RV/LV = right-to-left ventricular diameter ratio.

### Escalation and bailout strategies

Clinical deterioration requiring escalation of support occurred in 23 patients (18.0%). Thirteen patients showed insufficient CT response after the initial 24-h EKOS cycle and underwent a second 12-h EKOS cycle, of whom 8 responded and 5 required rescue surgical embolectomy; 3 survived and 2 died. Six patients required VA-ECMO during ongoing EKOS procedures. Of these, 4 were successfully weaned, whereas 2 required rescue surgical embolectomy; 1 survived and 1 died. No patient underwent systemic thrombolysis as bailout after EKOS. Details of institutional treatment allocation and escalation pathways are shown in **Figure 1**.

### Survival

**Figure 3.**
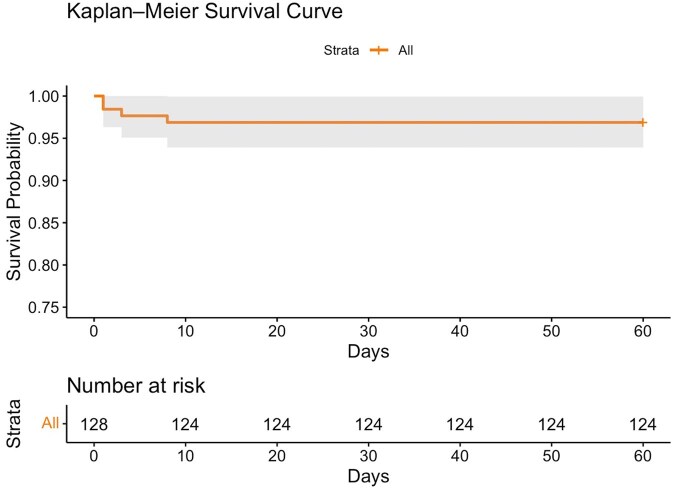
Kaplan–Meier Curve Showing 60-Day Survival Following EKOS Therapy. Short-term survival probability was favourable in the study cohort, with early events plateauing after the first days post-intervention.

Survival analysis was performed in all 128 patients. During 60 days of follow-up, four deaths were observed, corresponding to an overall mortality rate of 3.1%. Short-term survival remained favourable, with an estimated 60-day survival probability of 96.9% (**Figure 3**). Most adverse events occurred during index hospitalization and were concentrated in patients with greater baseline severity requiring escalation of support. Most deaths occurred within the first 10 days of hospitalization, with survival stabilizing thereafter.

## DISCUSSION

### Principal findings and right ventricular response

In this real-world PERT-guided cohort, EKOS was associated with rapid and consistent physiological improvement. However, early physiological improvement did not always translate into clinical stability. Significant reductions in RV/LV ratio were accompanied by parallel decreases in BNP and cardiac troponin, indicating effective RV unloading (**Figure 2**). The magnitude of RV/LV ratio reduction was comparable to that reported in the ULTIMA, SEATTLE II, and OPTALYSE-PE trials.^6–8^

### Biomarker trajectories and myocardial stress

Declines in BNP and troponin were consistent with early haemodynamic recovery and with their established prognostic role in acute PE.^16^^,^^17^ However, serial biomarker trajectories following EKOS have been less consistently reported outside controlled trials.

### Organ dysfunction and clinical deterioration beyond haemodynamic improvement

Early physiological improvement did not always translate into clinical stability. Respiratory and renal dysfunction were largely pre-existing at presentation and did not increase after EKOS, indicating that these complications reflected advanced baseline illness severity rather than procedural harm.^10^^,^^18^ These observations support the concept that acute PE represents a systemic disease with downstream multi-organ consequences, even when early reperfusion is achieved.^19^ RV unloading alone may be insufficient to fully mitigate the inflammatory, haemodynamic, and microcirculatory disturbances triggered by severe embolic burden.^19^^,^^20^

Our findings, in line with earlier reports,^10^^,^^11^ support the interpretation that these events reflected advanced baseline illness severity rather than procedural complications.

Consistent with this concept, we have previously shown that baseline systemic organ dysfunction as reflected by an abnormal De Ritis ratio is strongly associated with shock, multiorgan failure, and mortality in intermediate–high-risk PE.^21^

### Composite clinical endpoint and determinants of adverse outcome

To capture clinically relevant deterioration beyond mortality alone, we applied a composite end-point integrating death, ECMO, renal replacement therapy, and invasive mechanical ventilation. This approach reflects real-world clinical decision-making and acknowledges that escalation of organ support constitutes a meaningful adverse outcome in acute PE management.^9^^,^^22^^,^^23^

In multivariable analysis, baseline clinical severity as reflected by high-risk PE Severity Index classification, and acute kidney failure emerged as the dominant determinants of the composite end-point, whereas post-interventional RV/LV ratio did not retain independent prognostic significance. These findings are consistent with prior evidence demonstrating that baseline risk profile and early end-organ dysfunction are stronger predictors of adverse outcomes than isolated haemodynamic parameters.^1^^,^^19^^,^^24^ Accordingly, early physiological improvement should not be overinterpreted as a surrogate for clinical stability, particularly in patients with advanced baseline risk.

### Role of multidisciplinary PERT-Guided management

All patients were managed within a structured PERT framework integrating interventional cardiology, cardiac surgery, thoracic surgery, cardioanesthesiology, and pneumology expertise. Growing evidence suggests that PERT-based care may improve risk stratification, accelerates access to advanced reperfusion strategies, and standardizes multidisciplinary decision-making in complex PE cases.^13–15^^,^^25^ The favourable short-term survival observed in our cohort, despite a substantial burden of intermediate–high-risk features, should be interpreted in the context of this structured multidisciplinary pathway.^25^^,^^26^

### Ultrasound-Assisted vs conventional Catheter-Directed thrombolysis

Nevertheless, the reproducible physiological response and acceptable safety profile observed in our cohort suggest that EKOS may represent a feasible reperfusion option in carefully selected patients within structured care pathways, particularly when embedded within multidisciplinary PERT-guided decision-making.^14^^,^^15^

These findings indicate that EKOS was associated with favourable early survival, while subsequent escalation of care appeared to reflect underlying disease severity rather than procedural harm.

### Limitations

Several limitations merit consideration. The retrospective, single-centre design introduces potential selection bias and precludes causal inference. The absence of a contemporaneous control group limits assessment of the relative effect of EKOS compared with alternative reperfusion strategies or standard anticoagulation alone.

Imaging modality for RV/LV assessment was determined by clinical availability, and measurements were not centrally adjudicated. Event numbers limited multivariable modelling, and unmeasured confounding cannot be excluded. Long-term outcomes were not systematically captured.

## CONCLUSIONS

In this real-world PERT-guided cohort, EKOS was associated with rapid haemodynamic and biomarker improvement in patients with intermediate–high-risk PE. Despite favourable short-term survival, clinically meaningful in-hospital deterioration remained common and was associated primarily with baseline disease severity and early organ dysfunction rather than post-interventional haemodynamic response alone. These findings emphasize the need for comprehensive risk assessment beyond early RV unloading and should be interpreted as descriptive single-centre experience.

## Supplementary Material

ivag168_Supplementary_Data

## Data Availability

The data underlying this article will be shared on reasonable request to the corresponding author.

## Abbreviations

AKF: acute kidney failure
ALT: alanine aminotransferase
AST: aspartate aminotransferase
BNP: B-type natriuretic peptide
CI: confidence interval
COPD: chronic obstructive pulmonary disease
CT: computed tomography
ECMO: extracorporeal membrane oxygenation
EKOS: Eko Sonic Endovascular System
IQR: interquartile range
OR: odds ratio
PE: pulmonary embolism
PERT: pulmonary embolism response team
PESI: Pulmonary Embolism Severity Index
RV: right ventricle
RV/LV: right-to-left ventricular diameter ratio
USAT: ultrasound-assisted catheter-directed thrombolysis
