# Supplementary material for: Outcomes of EKOS (Ultrasound-Assisted Thrombolysis) for Intermediate–High-Risk Pulmonary Embolism: A PERT-Guided Cohort Study
Source: Interdiscip Cardiovasc Thorac Surg. 2026 Jun 2;41(6):ivag168. doi: 10.1093/icvts/ivag168 (PMC13278780; doi:10.1093/icvts/ivag168)
Supplement: ivag168_Supplementary_Data [file ivag168_supplementary_data.docx]

# Supplementary Table S1. Sensitivity subanalysis excluding baseline critical illness

Sensitivity analysis excluding patients with pre-intervention shock, mechanical ventilation, acute kidney failure, or pre-intervention mechanical circulatory support. Values are presented as n (%).

| Outcome | Full cohort (n=128) | Sub-cohort excl. baseline critical illness (n=96) |
| --- | --- | --- |
| Acute respiratory insufficiency | 63 (49.2%) | 43 (44.8%) |
| Acute hemorrhagic anemia | 28 (21.9%) | 11 (11.5%) |
| Cardiac arrest | 10 (7.8%) | 3 (3.1%) |
| Extracorporeal life support (ECLS) | 6 (4.7%) | 0 (0%) |
| Renal replacement therapy (Dialysis) | 7 (5.5%) | 0 (0%) |
| Acute kidney failure | 19 (14.8%) | 0 (0%) |
